# CO_2_ as a Redox Shuttle Enables Photocatalytic Dehydrogenative Decarbonylation of Biomass‐Derived Alcohols to Light Alkanes

**DOI:** 10.1002/anie.9277722

**Published:** 2026-07-24

**Authors:** Jun Hu, Wenhao Su, Chaoqin Zeng, Bruno Vinicius Manzolli Rodrigues, Nils Rockstroh, Susanna Monti, Giovanni Barcaro, Piotr Kuśtrowski, Aleksander Jaworski, Jabor Rabeah, Adam Slabon, Shoubhik Das

**Affiliations:** ^1^ Department of Chemistry University of Bayreuth Bayreuth Germany; ^2^ State Key Laboratory of Low Carbon Catalysis and Carbon Dioxide Utilization Lanzhou Institute of Chemical Physics Chinese Academy of Sciences Lanzhou Gansu China; ^3^ University of Chinese Academy of Science Beijing China; ^4^ Department of Inorganic Chemistry University of Wuppertal Wuppertal Germany; ^5^ Leibniz‐Institut für Katalyse e.V. (LIKAT Rostock) Rostock Germany; ^6^ CNR‐ICCOM, Institute of Chemistry of Organometallic Compounds Pisa Italy; ^7^ CNR‐IPCF, Institute for Chemical and Physical Processes Pisa Italy; ^8^ Faculty of Chemistry Jagiellonian University Kraków Poland; ^9^ Department of Chemistry Stockholm University Stockholm Sweden

**Keywords:** biomass alcohol, CO_2_ as redox shuttle, dehydrogenative decarbonylation, heterogeneous photocatalyst

## Abstract

The use of CO_2_ as a redox shuttle offers an emerging strategy to regulate electron and hydrogen transfer in catalytic transformations, yet its potential remains largely unexplored. In particular, dehydrogenative decarbonylation of alcohols possesses a long‐standing challenge, as it requires the controlled coupling of oxidative alcohol activation with reductive C─C bond cleavage, two intrinsically competing processes that are difficult to balance within a single catalytic system. Considering these, we demonstrate that CO_2_ can resolve this mismatch by functioning as a dynamic redox shuttle in a heterogeneous photocatalytic platform. Under visible‐light irradiation, the photocatalyst promotes sequential dehydrogenation of primary alcohols to aldehydes, followed by C─C bond scission and selective formation of alkanes. Mechanistic studies, including isotope labeling, radical trapping, atmosphere‐dependent reactivity, and advanced quantum mechanical calculations reveal that CO_2_ is not incorporated into the products but instead transiently interacts with reduced iron sites to facilitate catalyst turnover, suppress unproductive H_2_ evolution, and direct hydrogen equivalents toward C─H bond formation. This redox‐shuttling function enables the integration of oxidative and reductive steps within a single photocatalytic cycle, thus opening new opportunities for steering complex redox transformations in photocatalysis.

## Graphical abstract

A heterogeneous photocatalytic system has been developed that enables the dehydrogenative decarbonylation of primary alcohols under visible light using highly dispersed oxidic iron sites supported on aryl‐modified graphitic carbon nitride (Fe@f–gC_3_N_4_).

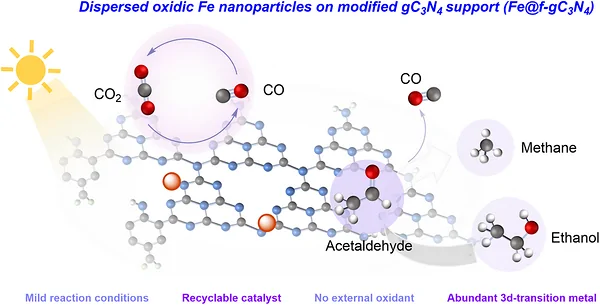

## 1 Introduction

Carbon dioxide (CO_2_) can function as a redox shuttle by reversibly accepting and donating electrons under catalytic conditions, thereby mediating electron transfer processes without being consumed stoichiometrically [1, 2, 3]. In this approach, CO_2_ either undergoes single‐electron reduction to generate the CO_2_^•−^ radical or two‐electron/proton‐coupled reduction to formate or carbon monoxide, which can subsequently be reoxidized to regenerate CO_2_, thereby closing a catalytic redox cycle. This bidirectional electron‐transfer capability resolves challenging redox transformations by transiently storing and releasing reducing equivalents, effectively decoupling substrate activation from direct electrode or photon‐driven processes [4, 5, 6]. For example, in molecular electrosynthesis, CO_2_‐derived intermediates have been shown to facilitate the reductive activation of inert bonds (e.g., C─Cl, C─O), stabilizing high‐energy radical or anionic species and enabling selective cross‐coupling and defunctionalization pathways [7, 8, 9]. Furthermore, in transition‐metal catalysis, reversible CO_2_ coordination can modulate the electronic structure and oxidation state of the metal center, functioning as a non‐innocent ligand that participates directly in redox events along the catalytic cycle [10, 11]. This concept is particularly powerful in paired electrolysis, where cathodic CO_2_ reduction is coupled with the anodic oxidation of organic substrates to improve overall faradaic efficiency and energy utilization [12, 13]. These approaches have been well documented in reductive carboxylation reactions, CO_2_‐mediated hydrogenation and transfer hydrogenation processes, as well as for the formation of carbon–carbon and carbon–heteroatom bonds under mild conditions [14, 15]. Along this direction, photoredox systems further exemplify CO_2_ as a transient electron reservoir, enabling access to highly reducing potential while maintaining catalyst stability and selectivity [16, 17]. Collectively, these advances position CO_2_ as a uniquely sustainable redox mediator that bridges fundamental electron‐transfer chemistry with practical applications in synthesis, catalysis, and energy conversion [18, 19].

**FIGURE 1:**
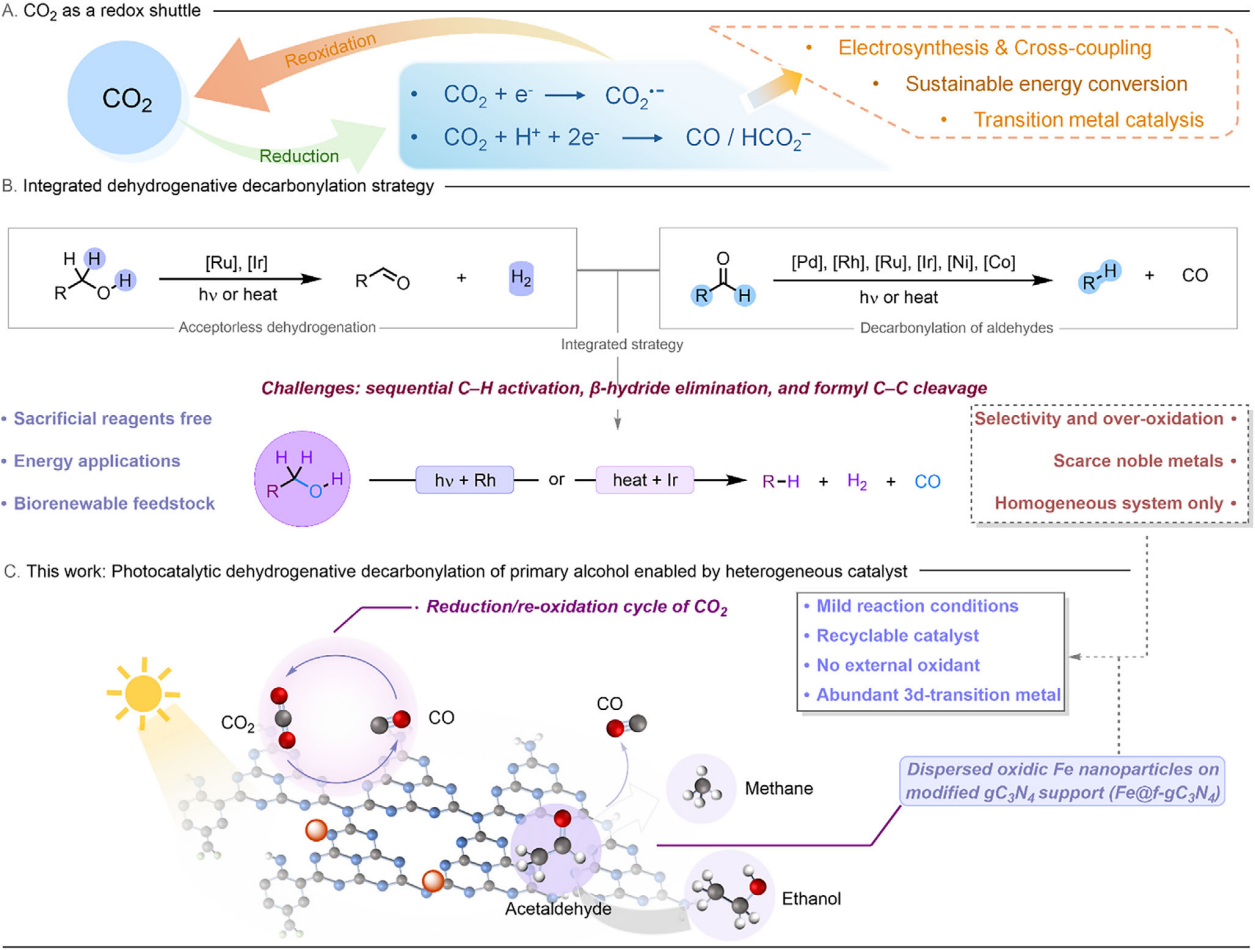
Integrated strategy for dehydrogenative decarbonylation of alcohols.

Dehydrogenation of alcohols and decarbonylation of aldehydes constitute two cornerstone transformations for both molecular upgrading and small‐molecule valorization [20, 21, 22, 23, 24]. In particular, catalytic alcohol dehydrogenation to carbonyl compounds, coupled with the evolution of molecular hydrogen (H_2_), has emerged as a pioneering approach for redox‐neutral substrate activation and sustainable H_2_ generation [25, 26, 27, 28]. In contrast, aldehyde decarbonylation, characterized by challenging C─C bond scission and CO extrusion, has predominantly been realized under forcing thermal conditions with rhodium‐, iridium, or palladium‐based catalysts (Figure 1B) [29, 30, 31]. Despite the maturity of these individual transformations, their integration into a single dehydrogenative decarbonylation sequence remains a formidable challenge to orchestrate sequential C─H activation, β‐hydride elimination, and formyl C─C bond cleavage while simultaneously tolerating strongly coordinating carbonyl and CO intermediates that can poison the active site of the transition metal complex [32, 33, 34]. Early breakthroughs in this direction were achieved in homogeneous systems, for example, Madsen and coworkers demonstrated that the [Ir(COD)Cl]_2_/BINAP system could affect the dehydrogenative decarbonylation of primary alcohols at elevated temperatures (160°C), liberating H_2_ and CO as syngas [35]. Furthermore, Sadow and coworkers reported a photochemical variant based on the rhodium complex [To^M^Rh(CO)_2_] (To^M^ = tris(4,4‐dimethyl‐2‐oxazolinyl)phenylborate), enabling the same transformation under UV irradiation at ambient temperature [32]. While this transformation can be achieved with substituted or long‐chain alcohols, application of this strategy towards lighter aliphatic alcohols such as ethanol and propanol becomes more complicated [32, 36]. In such cases, the absence of stabilizing substituents renders both the intermediate aldehydes (e.g., acetaldehyde and propionaldehyde) and the corresponding acyl species highly reactive and susceptible to undesired side reactions, including overoxidation, aldol condensation, and complete decomposition [37, 38]. Moreover, the formation of strongly bound CO from rapid decarbonylation can exacerbate catalyst deactivation, particularly under heterogeneous conditions [39, 40, 41, 42]. Nonetheless, if this transformation is achieved, this strategy should enable the production of highly valuable lighter alkanes such as methane, ethane, pentane, butane and many others from renewable biomass‐derived lower aliphatic alcohols [43, 44]. In this context, the global market value of methane (natural gas) was estimated at around USD 350 billion in 2025, while individual gas markets for ethane (∼USD 10–15 billion), propane (∼USD 120–130 billion), and butane (∼USD 70–90 billion) reflected their distinct roles as fuels and petrochemical feedstocks. Looking ahead, demand for all these four gases is expected to increase steadily (≈ 3%–8% CAGR) (CAGR = compound annual growth rate), with methane driven by energy transition needs, and ethane, propane, and butane seeing strong growth from expanding petrochemical production, LPG consumption, and industrial applications.

Intrigued by the above information, we set out to design an iron‐based heterogeneous photocatalyst to produce lighter alkanes from biomass‐derived aliphatic alcohols via a single‐step dehydrogenation‐decarbonylation process, in which CO_2_ should serve as a redox shuttle. We argued that integrating an iron‐based photocatalyst supported on functionalized graphitic carbon nitride (f‐gC_3_N_4_) would offer a compelling strategy to overcome the long‐standing challenges associated with the dehydrogenative decarbonylation of lower aliphatic alcohols. In our approach, upon visible‐light irradiation, f‐gC_3_N_4_ should generate long‐lived charge‐separated states enabling efficient photoredox activation of both the alcohol and the catalytic iron centers. The iron sites should facilitate sequential C–H activation and β‐hydride elimination, while their heterogeneous immobilization will suppress aggregation and enhance the catalyst stability [45, 46, 47, 48, 49, 50, 51]. Crucially, the incorporation of CO_2_ as a redox shuttle should enable reversible electron storage and transfer, thereby decoupling oxidative dehydrogenation from reductive C─C bond cleavage within a single catalytic cycle [11, 52, 53]. In this context, the formation of transient species from CO_2_ will buffer photogenerated electrons, preventing charge recombination and mitigating overreduction of reactive intermediates [54, 55]. The cooperative interplay between iron active sites, photoexcited f‐gC_3_N_4_, and CO_2_‐mediated electron transfer should thus enable a balanced catalytic regime that will promote both dehydrogenation and decarbonylation steps with improved selectivity.

## 2 Results and Discussion

### 2.1 Synthesis of the Photocatalyst

Dispersed iron nanoparticles supported on aryl‐modified graphitic carbon nitride (Fe@f–gC_3_N_4_) were synthesized *via* a two‐step impregnation–calcination procedure without the use of a template (see Supporting Information) [56, 57, 58]. In a typical synthesis, dicyandiamide (DCDA) and 2‐amino‐5‐(trifluoromethyl)benzonitrile were dissolved in water, followed by the addition of Fe(NO_3_)_3_·9H_2_O as the iron precursor. The resulting mixture was stirred and heated to 95°C–100°C until the solvent was completely evaporated. The obtained solid residue was finely ground, transferred to a stainless‐steel crucible, and calcined in air at 550°C. After cooling to room temperature, the product was collected and ground to afford the final photocatalyst. To disentangle the individual contribution of iron incorporation and aryl modification, three reference materials were prepared under identical conditions. First, iron‐doped unmodified graphitic carbon nitride (Fe@g‐C_3_N_4_) was synthesized in the absence of the aryl co‐ligand. Second, aryl‐modified g‐C_3_N_4_ without iron (f‐gC_3_N_4_) and third, pristine g‐C_3_N_4_ were obtained by omitting the metal precursor as well as by excluding both the metal precursor and the aryl co‐ligand, respectively. This series of materials enabled a systematic assessment of the structural, electronic, and photocatalytic roles of iron incorporation and aryl modification. The electronic band structure of Fe@f‐gC_3_N_4_ was characterized *via* UV–vis diffuse reflectance spectroscopy (DRS) and Mott–Schottky analysis (Figures S4 and S5). Tauc plot extrapolation yielded an optical bandgap of 2.50 eV. The positive slope of the Mott–Schottky plot confirmed n‐type semiconducting behaviour, allowing the conduction band potential (*E*_cb_) to be approximated from the flat‐band potential (*E*_fb_). Specifically, *E*_cb_ was determined to be −0.43 V (vs. RHE), placing the valence band edge (*E*_vb_) at approximately +2.07 V (vs. RHE).

### 2.2 Optimization of Reaction Conditions

**FIGURE 2:**
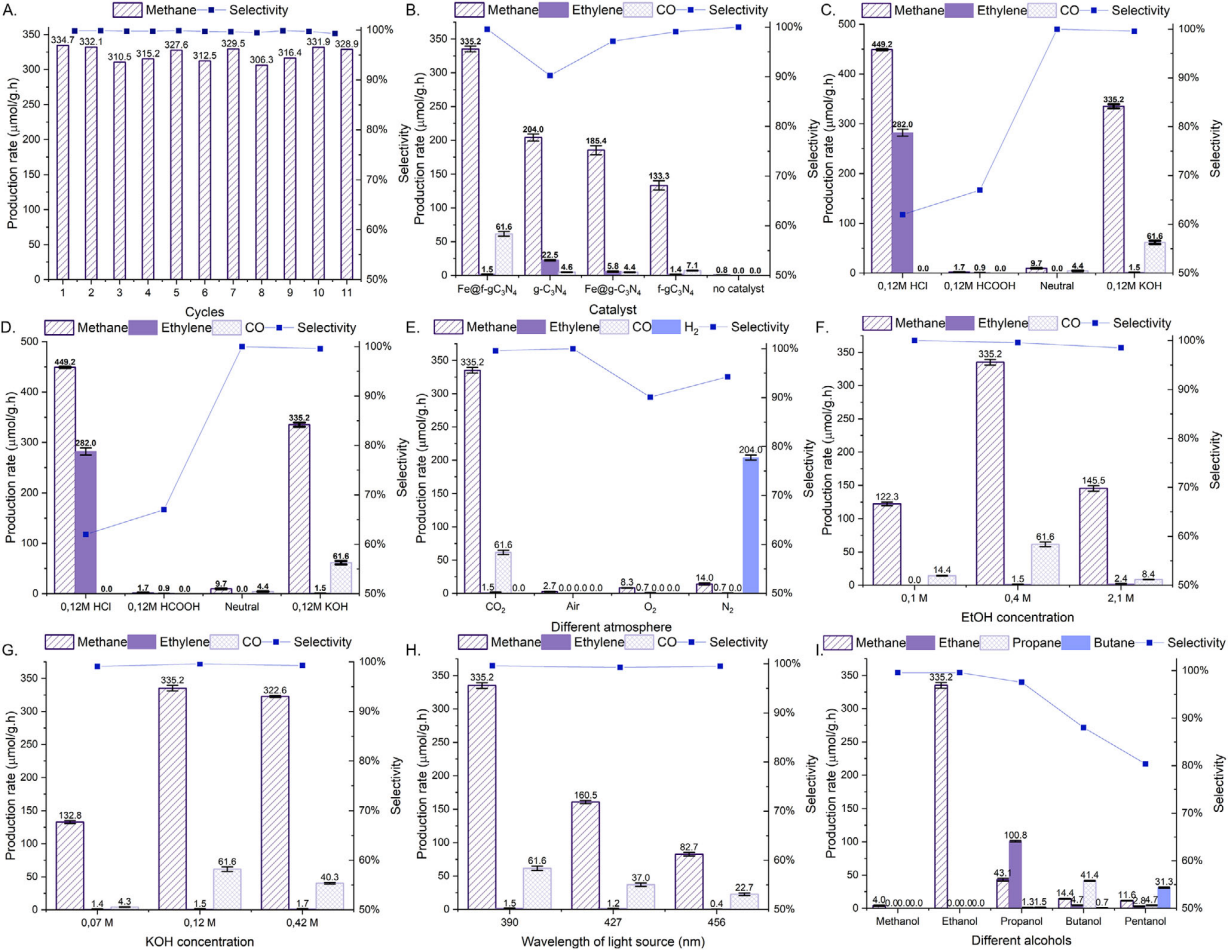
Photocatalytic performance and optimization of the Fe@f‐gC_3_N_4_ photocatalyst. (A) Recyclability experiments for the dehydrogenative decarbonylation of ethanol. Standard conditions: 4 mg photocatalyst (PC), 0.12 M KOH, 0.1 mL alcohol, 4 mL acetonitrile, 390 nm, 18 h under CO_2_ atmosphere. (B) Activity comparison of ethanol decarbonylation using various g‐C_3_N_4_‐based catalysts. Optimization of reaction parameters using Fe@f‐gC_3_N_4_, including (C) pH levels, (D) base additives, (E) reaction atmospheres, (F) ethanol concentration, (G) KOH concentration, and (H) incident light wavelength. (I) Scope of the photocatalytic dehydrogenative decarbonylation of different lighter alcohols.

After the synthesis and photophysical studies of the iron‐based photocatalysts, the photocatalytic dehydrogenative decarbonylation of ethanol was evaluated in acetonitrile with KOH as a base under a CO_2_ atmosphere (balloon) upon visible‐light irradiation (*λ*_max_ = 390 nm). To our delight, when Fe@f‐gC_3_N_4_ was examined under the following reaction conditions (4 mg of photocatalyst, 0.12 M of KOH, 0.1 mL of ethanol, and 4 mL of acetonitrile), methane was obtained as the dominant gaseous product with a formation rate of 334.7 µmol g^−1^ h^−1^ (corresponding to a methane selectivity of 98%), accompanied by 61.3 µmol g^−1^ h^−1^ of CO and 1.4 µmol g^−1^ h^−1^ of ethene. Consistent with a sequential alcohol dehydrogenation–decarbonylation pathway, acetaldehyde was identified as the primary liquid‐phase product by GC–FID, which was further validated by ^1^H and ^13^C NMR spectroscopy. Control experiments confirmed that no product was formed in the absence of ethanol, light irradiation, or the photocatalyst, establishing that all these components were essential for photocatalytic methane formation (Table S1). Furthermore, the solvent proved critical for catalytic performance; for example, when ethanol was used as the neat solvent in the absence of acetonitrile, a lower methane formation rate (115 µmol g^−1^ h^−1^) was observed. This may be attributed to the role of acetonitrile in stabilizing reactive intermediates, facilitating interfacial charge transfer, and enhancing CO_2_ solubility [59, 60]. Additionally, under CO_2_‐buffered conditions, ethanol likely acted as a protic solvent capable of forming hydrogen bonds with the basic nitrogen sites of f‐gC_3_N_4_. In contrast, acetonitrile, being aprotic, did not block these sites as effectively, allowing the photocatalyst to maintain higher activity for alcohol dehydrogenation [61, 62]. Moreover, if ethanol underwent self‐cleavage, the resulting intermediates (e.g., ethoxy species or CO/methane byproducts) might act as catalytic poisons or occupy active sites required for C─C bond cleavage in larger, more complex alcohols [63]. Following these observations, a comparison with other photocatalysts (bare g‐C_3_N_4_, Fe@g‐C_3_N_4_, and f‐gC_3_N_4_) was performed, yielding 204.8, 187.9, and 135.8 µmol g^−1^ h^−1^ of methane formation, respectively (Figure 2B). We attributed the superior performance of Fe@f‐gC_3_N_4_ to the synergistic combination of redox‐active iron sites, which facilitated alcohol oxidation and C─C bond activation, and the aryl‐modified g‐C_3_N_4_ scaffold, which enhanced charge separation and interfacial electron transport required for coupling oxidative and reductive half‐reactions [64].

Encouraged by the above information, we investigated the effect of the base on methane formation, as the reaction medium exerted a decisive influence on both reactivity and product selectivity. Under neutral conditions, methane formation dropped sharply to 9.7 µmol g^−1^ h^−1^ (Figure 2C), highlighting the critical role of a basic environment in enabling efficient proton‐coupled electron transfer and activating the catalytic sites required for this multistep reduction process. This was followed a systematic investigation of base, which further underscored this effect, and identified KOH (0.12 M) as the optimal choice. Indeed, the observed reactivity trend (Cs^+^ > K^+^ > Na^+^ > Li^+^) aligned well with the established correlation between cation polarizability and CO_2_ adsorption/activation at semiconductor interfaces (Figure 2D) [65, 66]. We rationalized that the highly alkaline conditions introduced by KOH promoted the strong coordination of OH^−^ anions at the Fe catalytic sites, alongside the potential base‐catalyzed hydrolysis of terminal amino defects at the g‐C_3_N_4_ edges. This localized accumulation of surface hydroxyl groups modulated the electronic structure of the photocatalyst, thereby enhancing its redox capability and facilitating charge transfer kinetics [67]. Additionally, under CO_2_‐buffered conditions, the presence of K^+^ ions in a basic medium promoted the formation of bicarbonate/carbonate species, which stabilized key surface‐bound intermediates (e.g., CO* or COOH*) and suppressed their premature desorption as CO [68, 69]. In parallel, the alkaline environment regulated the local proton availability and improved charge‐carrier separation, collectively steering the reaction pathway toward methane formation [70]. Although CsOH delivered methane formation rates comparable to those of KOH, its practical utility is limited by its higher cost and stronger catalyst–surface interactions, which compromise recyclability. In contrast, carbonate bases such as K_2_CO_3_ and Cs_2_CO_3_ exhibited only moderate reactivity, whereas NaOH and LiOH resulted in significantly diminished methane yields [57]. Notably, acidic conditions induced a pronounced mechanistic shift: in 0.12 M of HCl, methane formation increased to 449 µmol g^−1^ h^−1^, however, ethene emerged as the dominant product (278 µmol g^−1^ h^−1^), indicating that proton‐rich environments preferentially promoted dehydration pathways over decarbonylation [71]. In contrast, formic acid nearly suppressed all photocatalytic reactivity, likely due to competitive proton reduction and concomitant catalyst deactivation [72].

The reaction atmosphere was equally critical (Figure 2E). Under CO_2_ and basic conditions, methane was the exclusive hydrocarbon product, while the hydrogen evolution reaction (HER) was effectively suppressed. The hydrogen equivalents generated during alcohol dehydrogenation were therefore likely consumed in proton‐coupled surface processes, plausibly forming water as the terminal hydrogen sink, consistent with a redox‐balanced photocatalytic cycle [73]. In stark contrast, reactions conducted under N_2_ atmosphere yielded predominantly H_2_ (202 µmol g^−1^ h^−1^) with only trace methane (14.7 µmol g^−1^ h^−1^). Similarly, experiments under air or O_2_ exhibited negligible reactivity, emphasizing the indispensable, albeit indirect, role of CO_2_ in stabilizing reduced iron sites and suppressing unproductive hydrogen recombination pathways [74]. Finally, the interplay between substrate and base concentrations was systematically examined. An optimal ethanol concentration of 0.4 M was identified (Figure 2F); lower concentrations limited surface coverage, whereas higher concentrations impeded oxidation due to competitive adsorption [75]. Likewise, 0.12 (M) KOH proved to be optimal (Figure 2G), with lower alkalinity diminishing reactivity, and higher base loadings slightly increasing CO formation without improving the methane yield. Furthermore, photocatalytic performance also showed a strong dependence on excitation wavelength (Figure 2H), closely following the absorption profile of Fe@f‐gC_3_N_4_. Illumination at 427 nm produced 160 µmol g^−1^ h^−1^ of CH_4_, while excitation at 456 nm yielded 82 µmol g^−1^ h^−1^, confirming that activation of the charge‐transfer band was essential to drive the photocatalytic process. Encouraged by these results, the best‐performing photocatalyst (Fe@f‐gC_3_N_4_) was evaluated over more than 10 consecutive cycles and, notably, showed no significant loss in activity, demonstrating excellent stability (Figure 2A).

The scope of the photocatalytic dehydrogenative decarbonylation was subsequently explored by using a range of lighter alcohols under the optimized conditions (Figure 2I). Methanol produced only trace amounts of methane, consistent with the substantial energetic barrier associated with its initial dehydrogenation step. In contrast, other alcohols underwent efficient transformation, selectively yielding one‐carbon‐shorter alkanes as the dominant hydrocarbon products. For instance, 1‐propanol generated CH_4_ and C_2_H_6_ at formation rates of 43.1 and 100.8 µmol g^−1^ h^−1^, respectively, while 1‐butanol afforded methane (14.4 µmol g^−1^ h^−1^) and propane (41.4 µmol g^−1^ h^−1^). Similarly, 1‐pentanol delivered methane (11.6 µmol g^−1^ h^−1^) alongside *n*‐butane (31.3 µmol g^−1^ h^−1^). This consistent trend strongly supported a general mechanistic pathway involving sequential alcohol dehydrogenation followed by C─C bond cleavage, ultimately producing alkanes with one fewer carbon atom, and demonstrated that Fe@f‐gC_3_N_4_ maintained high photocatalytic competence across medium‐chain substrates. In this context, longer chain alcohols failed to produce the corresponding alkane in high yield due to the combined effects of reduced O─H acidity from electron‐donating alkyl groups, increased steric hindrance, and conformational flexibility, which hinder effective catalyst interaction and β‐H elimination [76, 77]. Polyfunctional alcohols exhibited markedly reduced reactivity as well. Ethylene glycol and glycerol yielded only trace quantities of methanol (0.7 µmol g^−1^ h^−1^) and ethylene glycol (0.5 µmol g^−1^ h^−1^), respectively, indicating that while dehydrogenative decarbonylation remained feasible, it was significantly impeded by competing coordination effects and parallel oxidation pathways that limited efficient C─C bond scission [78]. To further probe the robustness and practical relevance of the system, Fe@f‐gC_3_N_4_ was evaluated under mixed‐substrate conditions. When a 1:1 mixture of ethanol and propanol was employed, methane (111.7 µmol g^−1^ h^−1^) and ethane (33.8 µmol g^−1^ h^−1^) emerged as the principal products. Likewise, a mixture of butanol and pentanol produced appreciable amounts of propane (36.4 µmol g^−1^ h^−1^) and *n*‐butane (13.2 µmol g^−1^ h^−1^). The efficient formation of propane and butane under mild, visible‐light‐driven conditions highlighted the capacity of this photocatalytic system to process complex, renewable alcohol feeds into valuable light hydrocarbons. In summary, these findings established that Fe@f‐gC_3_N_4_ enabled the efficient coupling of alcohol oxidation with selective C─C bond cleavage across a diverse substrate range under CO_2_‐buffered conditions. The reaction proceeded *via* a redox‐balanced photocatalytic dehydrogenative decarbonylation pathway, providing a versatile platform for transforming oxygenated feedstocks into value‐added alkanes under visible light irradiation.

### 2.3 Characterization of the Photocatalyst

**FIGURE 3:**
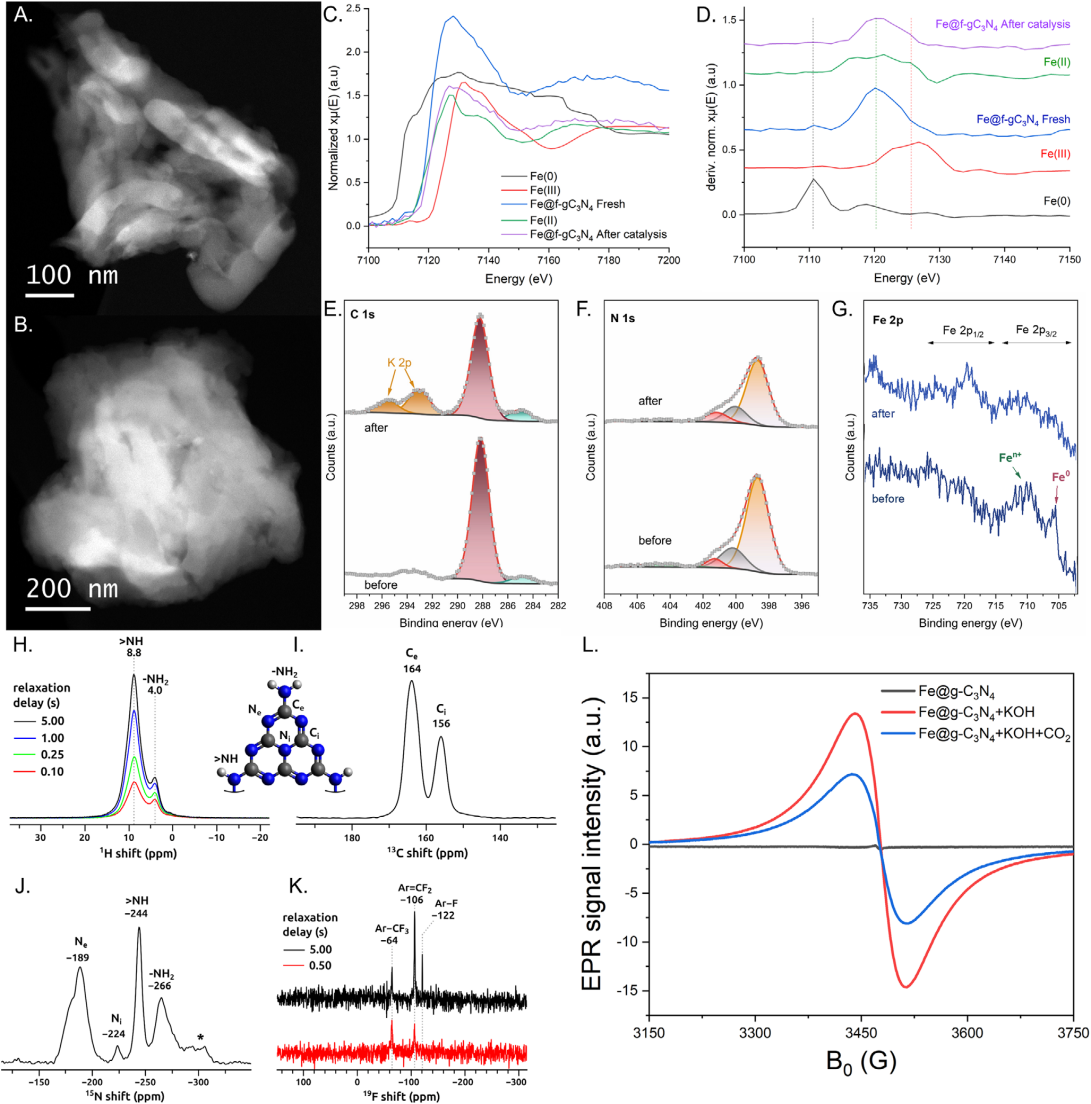
Catalyst characterizations. STEM‐HAADF images of Fe@f‐gC_3_N_4_ (A) before and (B) after catalysis. (C) Normalized Fe K‐edge XANES spectra of fresh and post‐catalysis Fe@f‐g‐C_3_N_4_ together with Fe foil (Fe^0^), FeNCN (Fe^2+^), and Fe_2_(SO_4_)_3_ (Fe^3+^) standards (*E*_0_ = 7112.0 eV). (D) First‐derivative XANES spectra used to assess relative edge positions and oxidation states. High‐resolution XPS spectra of Fe@f‐gC_3_N_4_ before and after photocatalysis in the C 1s (E), N 1s (F), and Fe 2p regions (G). Solid‐state ^1^H MAS (H), ^13^C CPMAS (I), ^15^N CPMAS (J), and ^19^F MAS (K) spectra collected from the Fe@f‐gC_3_N_4_ catalyst. Spinning sideband in panel J is marked with an asterisk. (L) First‐derivative EPR spectra of Fe@f‐gC_3_N_4_ catalyst under various conditions.

The morphology and structural stability of the photocatalyst were evaluated *via* scanning transmission electron microscopy (STEM). As shown in Figure 3A,B, iron nanoparticles were occasionally found across the aryl‐modified g‐C_3_N_4_ support. Energy‐dispersive x‐ray (EDX) spectroscopy (Figures S2 and S3) demonstrated spatially correlated Fe and O signals in both fresh and spent catalysts, confirming the retention of an oxidic environment. This structural integrity was further corroborated by Fe K‐edge x‐ray absorption near‐edge spectroscopy (XANES) (Figure 3C), where the absorption edge was shifted to higher energy relative to the Fe foil. It lacked spectral features characteristic of metallic Fe, thereby excluding the formation of bulk Fe^0^. Instead, the edge position and white‐line intensity lie between those of Fe^2+^ and Fe^3+^ references, consistent with a mixed‐valent oxidic state. In fact, first‐derivative analysis (Figure 3D) placed the edge inflection closer to Fe^2+^, indicating its predominance alongside a measurable Fe^3+^ fraction. Notably, after catalysis, a subtle but reproducible shift toward higher energy was observed, indicative of partial oxidation of surface‐accessible iron sites rather than a bulk phase transformation, highlighting the dynamic yet stable nature of the active species.

X‐ray photoelectron spectroscopy (XPS) further confirmed the preservation of the carbon nitride framework and its surface chemistry. The N 1s spectrum (Figure 3F) displayed distinct components at 398.7, 400.2, and 401.3 eV, attributed to triazine nitrogen (C─N═C), bridging N in N─(C)_3_ units, and terminal amino groups, respectively, along with a weak π–π* shake‐up feature at ca. 404.7 eV [79, 80]. Consistently, the C 1s spectrum (Figure 3E) was dominated by a peak at 288.2 eV corresponding to sp^2^‐hybridized carbon in N─C═N motifs, with only a minor contribution at 284.8 eV arising from adventitious carbon [81]. The derived N/C ratio of 1.41 closely matched the ideal stoichiometry of g‐C_3_N_4_, reinforcing the structural fidelity of the support. Despite the inherently weak Fe 2p signal (Figure 3G), resulting from the low Fe loading and its high dispersion on the support, analysis of this region suggests the presence of two Fe 2p_3/2_ contributions at 706.6 and 710.5 eV [82], attributable to reduced and oxidized Fe species, respectively. The estimated Fe^0^:Fe^n+^ ratio of approximately 2:3 indicates that oxidized iron species predominate on the catalyst surface. Importantly, the post‐reaction spectra remained largely unchanged, except for the appearance of K 2p signals at 293.1 and 295.5 eV, which could be attributed to the adsorption of potassium species from the alkaline reaction medium. This further indicates that the photocatalyst framework and active sites remained chemically stable throughout the photocatalytic process.

Solid‐state NMR spectra of all NMR‐active nuclei present in the material (^1^H, ^13^C, ^15^N, and ^19^F) were collected to confirm the chemical structure of the functionalized graphitic carbon nitride, and the corresponding spectra are shown in Figure 3H–K. The ^1^H MAS, ^13^C CPMAS, and ^15^N CPMAS spectra (Figure 3 panels H–J) exhibited a complete set of characteristic signals expected from polymeric carbon nitride. The material exhibited a very high polymerization degree, as indicated by the signal intensity ratio between >NH linkers and ‐NH_2_ groups. It can be concluded that the overall chemical structure and polymerization degree in this material were very similar to those of related f‐gC_3_N_4_ catalysts reported by us previously [57, 58]. Functionalization of the gC_3_N_4_ precursor was evidenced by the ^19^F MAS NMR spectra (Figure 3K). Observed ^19^F NMR signals at −64, −106, and −122 ppm indicated the existence of Ar−CF_3_, Ar═CF_2_, and Ar−F moieties in the material, and the Ar═CF_2_ group was the most abundant. Electron paramagnetic resonance (EPR) spectroscopy (Figure 3L) provided additional insight into the electronic structure of the iron sites. The fresh catalyst exhibited only a weak gC_3_N_4_ signal and no detectable Fe‐centered resonance, consistent with EPR‐silent high‐spin Fe species in oxidic clusters. Upon addition of KOH, the EPR intensity increased markedly, indicating modification of the local electronic and magnetic environment of the Fe sites. Complementary XPS and Fe K‐edge XANES measurements performed on a KOH‐treated sample showed no detectable change in the average Fe oxidation state, suggesting that the EPR response does not originate from Fe redox chemistry. Instead, the observed changes are consistent with alterations in ligand‐field symmetry and/or Fe─O─Fe magnetic interactions induced by hydroxide species. Subsequent introduction of CO_2_ led to a decrease in signal intensity, indicating further modification of the Fe environment, potentially through CO_2_‐derived surface coordination. These observations demonstrate that KOH and CO_2_ reversibly influence the local electronic structure of the oxidic Fe species under reaction‐relevant conditions.

### 2.4 Mechanistic Investigations

**FIGURE 4:**
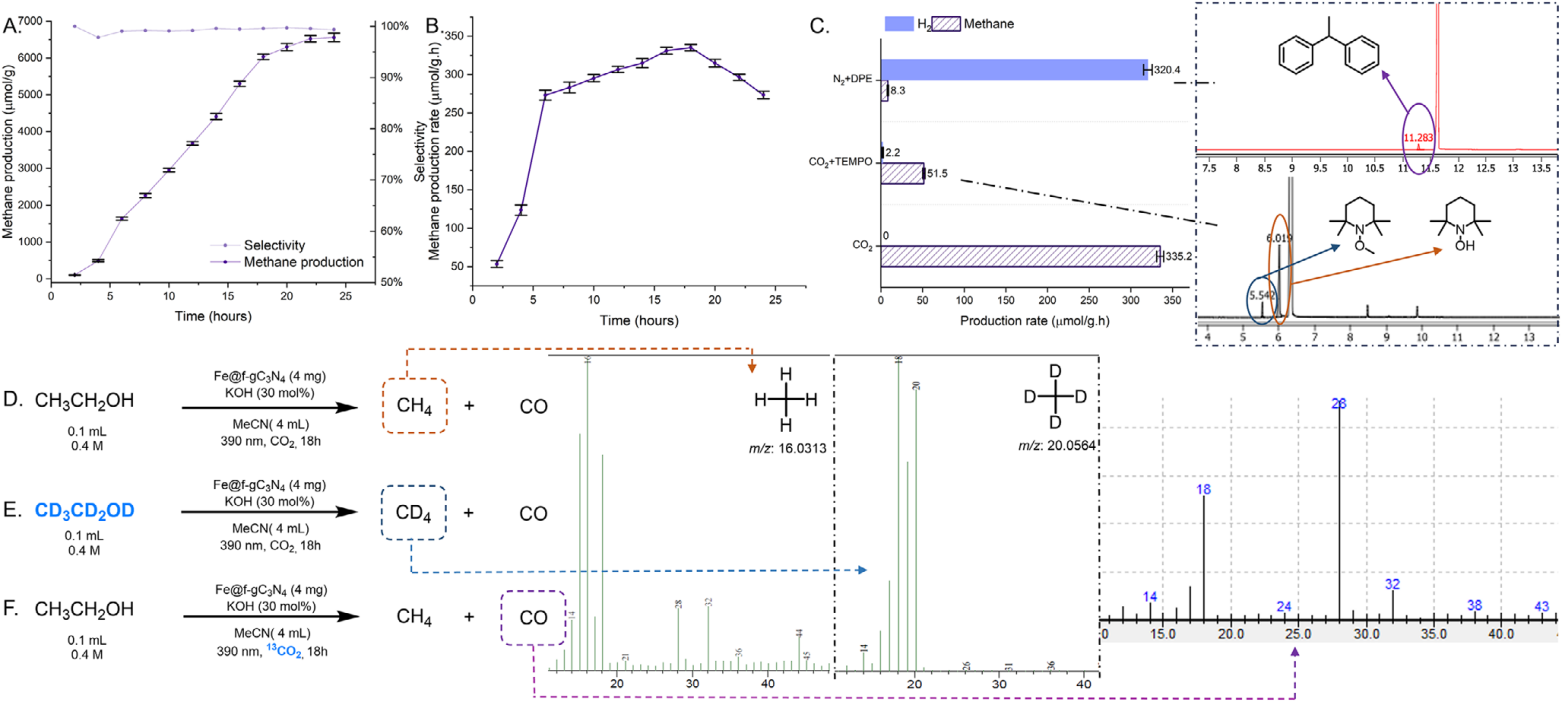
Mechanistic and kinetic studies of ethanol dehydrogenative decarbonylation. Standard conditions: photocatalyst (PC, 4 mg), KOH (0.12 M), alcohol (0.1 mL), acetonitrile (4 mL), 390 nm, 18 h, under CO_2_ atmosphere. (A, B) Reaction kinetic profiles for the photocatalytic dehydrogenative decarbonylation of ethanol using the Fe@f‐gC_3_N_4_ catalyst. (C) Product distribution and radical trapping experiments conducted under various atmospheres. Head‐space GC‐MS analysis of the products derived from the photocatalytic dehydrogenative decarbonylation of (D) ethanol, (E) deuterated ethanol, and (F) ethanol under a ^13^CO_2_ atmosphere.

To elucidate how oxidic iron nanoparticles dispersed on f‐gC_3_N_4_ promoted the photocatalytic dehydrogenative decarbonylation of primary alcohols, combined kinetic analysis, product identification, isotope labelling, and radical‐ and charge‐quenching experiments were performed. At first, time‐resolved product analysis indicated that methane formation proceeded rapidly during the initial hours of irradiation, followed by a slower increase up to ∼18 h, after which it reached a plateau (Figure 4A,B). This was consistent with the efficient early‐stage turnover and gradual catalyst deactivation due to surface accumulation of intermediates or substrate depletion [83]. Further, analyses of the liquid phase revealed that ethanol and acetaldehyde were the only detectable species, with no evidence of acetic acid formation, thereby excluding a decarboxylation pathway. Notably, the formation of acetaldehyde was strongly suppressed under an O_2_ atmosphere, indicating that oxidative quenching disrupted the formation or persistence of the key aldehyde intermediate required for subsequent C─C bond scission. In situ EPR spin‐trapping experiments using DMPO (5,5‐dimethyl‐1‐pyrroline *N*‐oxide) provide direct evidence for radical intermediates (Figure S11). Upon irradiation, a characteristic signal at *g* = 2.006 with hyperfine splitting constants *a*_N_ = 14.95 G and *a*_H_ = 22.32 G is observed, corresponding to a DMPO‐R• adduct. No additional radical signals were detected. These parameters were consistent with trapping of a carbon‐centered radical and strongly supported homolytic C─C bond cleavage following aldehyde formation. Later, radical‐trapping experiments further delineated the reactive intermediates involved in this reaction. The addition of TEMPO markedly suppressed methane formation and led to the detection of TEMPO‐methyl and TEMPO‐H adducts, directly evidencing the intermediacy of methyl radicals and hydrogen‐atom transfer processes (Figure 4C). This trend was further supported by experiments using diphenylethene (DPE) as a hydrogen acceptor, which was efficiently reduced under N_2_ atmosphere, concomitant with the formation of H_2_ detected by GC‐MS. On the other hand, in the absence of CO_2_, substantial evolution of H_2_ was observed, indicating that surface‐bound hydrogen species were generated during alcohol oxidation and preferentially recombined when they were not engaged in downstream reduction steps. Consistently, diphenylethene served as an efficient hydrogen acceptor under N_2_ atmosphere, undergoing reduction with concomitant H_2_ formation. These results established that H_2_ was generated and efficiently mobilized on the catalyst surface, with its fate determined by the reaction atmosphere.

Isotope‐labeling experiments unambiguously identified the origin of methane. To our observation, fully deuterated ethanol yielded a distribution of CD_x_Hᵧ isotopologues which were indicative of hydrogen scrambling and confirmed that methane was derived directly from alcohol (Figure 4E). In contrast, unlabeled ethanol produced CH_4_ exclusively (Figure 4D). Crucially, reactions conducted under a ^13^CO_2_ atmosphere did not exhibit any incorporation of ^13^C‐labeled carbon into either methane or in CO, demonstrating that both gaseous products originated exclusively from the alcohol (Figure 4F). This conclusion was further corroborated using propanol, which similarly produced unlabeled methane under ^13^CO_2_. These findings established that the cleavage of the C─C bond in alcohol‐derived aldehydes was the sole source of gaseous carbon‐containing products. The origin of H_2_ in methane was proved in complementary experiments. For example, neither D_2_O/NaOD nor deuterated acetonitrile (MeCN‐d_3_) led to deuterium incorporation into methane. These results demonstrated that neither the solvent nor the basic medium served as a hydrogen source and that ethanol itself acted as the primary hydrogen reservoir during both the oxidative and reductive steps of the reaction. Headspace GC–MS analyses under ^13^CO_2_ atmosphere provided additional insight, which revealed minor isotopic scrambling between CO_2_ isotopologues. Given the absence of ^13^C incorporation into products, this observation pointed to a reversible, surface‐mediated CO_2_/CO redox cycle at iron sites, involving transient formation and reoxidation of CO intermediates. Such dynamics are consistent with CO_2_ functioning as a redox shuttle that stabilizes catalytically competent iron oxidation states [54]. In this context, although CO_2_ was not incorporated into the products, it exerted a decisive influence on reaction selectivity. To our observation, in its absence, hydrogen evolution dominated, whereas under a CO_2_ atmosphere, methane formation became the preferred pathway. This switch indicated that CO_2_ regulated the fate of photogenerated electrons and surface hydrogen species rather than acting as a stoichiometric reactant. This is supported by the fact that CO_2_ transiently interacts with reduced iron centers, facilitating their reoxidation following ethanol activation and thereby maintaining the redox balance required for sustained catalysis [10]. In fact, this role was not replicated by conventional electron scavengers. At the same time, benzoquinone suppressed H_2_ evolution under N_2_, it did not promote methane formation, demonstrating that selective formation of C_1_ product required more than simple electron removal [54]. In addition, ex situ Fourier‐transform infrared (FTIR) measurements (Figure S15) provide complementary evidence for the involvement of CO_2_‐derived surface species. The catalyst recovered from the reaction mixture exhibits characteristic carbonate/bicarbonate/carbamate‐related vibrations, confirming the adsorption and activation of CO_2_ on the catalyst surface. Under alkaline conditions, the disappearance of bicarbonate/carbamate features and the emergence of a carbonate‐related band at 835 cm^−^^1^ suggest the formation of transient CO_2_‐derived intermediates. Concurrent attenuation of the ─NH_2_ stretching bands indicates the participation of surface amino groups in CO_2_ activation, likely through carbamate formation. These observations, together with the EPR results, support the interaction of CO_2_‐derived species with the Fe active sites and are consistent with the proposed CO_2_‐mediated redox‐shuttle mechanism.

**FIGURE 5:**
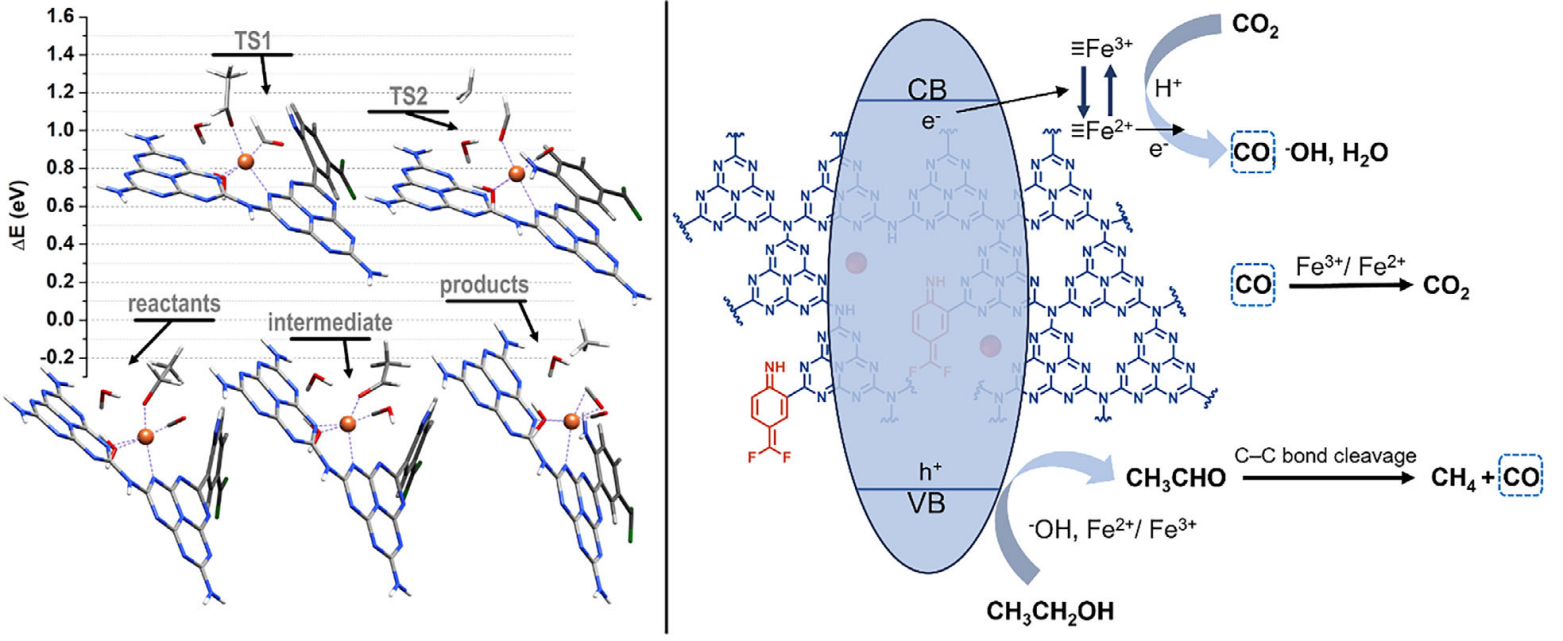
Proposed reaction scheme for photocatalytic dehydrogenative decarbonylation of primary alcohols.

To clarify the mechanism at the atomic‐electronic scale, we employed a simplified Fe‐N_2_ coordination model (Figure S6) to represent the photocatalytic cascade converting ethanol into methane (Figure 5, left). This configuration effectively captured the local electronic environment where critical bond‐breaking occurred, providing a reliable basis for calculating energy barriers as supported by recent literature [64]. The process began with CO_2_ activation, in which photoexcitation, modeled as an H^+^/e^−^ pair transfer, induced charge flow into the CO_2_ antibonding orbitals, leading to C─O bond elongation and O─C─O angle bending as the molecule was adsorbed onto the iron center in the form of COOH. The resulting fragments, primarily CO, ─OH, and H_2_O, remained adsorbed or localized nearby. Ethanol then entered the cycle by adsorbing onto the Fe active site *via* its oxygen atom in a deprotonated state. The released hydrogen may have combined with CO_2_‐derived OH to form water, been transferred to the catalyst's nitrogen framework, or contributed to experimentally observed H_2_ evolution. Photoexcitation subsequently triggered the rate‐limiting step, the selective dehydrogenation of the α‐C─H bond (TS1), which had an energy barrier of 1.4 eV. This step transferred a hydrogen atom to the adsorbed CO, forming a formyl radical (•CHO) while converting ethanol into the adsorbed acetaldehyde. This configuration evolved into a stable intermediate state (‐0.1 eV relative to reactants), in which the hydrogen on the formyl species migrated to its oxygen atom, forming an adsorbed CO─H fragment. Crucially, this state was stabilized by hydrogen bonding with the ‐NH group of the vicinal aryl co‐ligand, which trapped the molecule long enough for specific C─C cleavage to occur. The second transition state (TS2, 1.3 eV) represents this critical C─C methyl cleavage. The fact that TS2 was slightly lower in energy than TS1 suggested that once acetaldehyde was formed, the subsequent cleavage was relatively efficient. The synergy between the iron activation site and the orienting aryl moiety facilitated the detachment and rearrangement of oxygen‐containing species, yielding CH_4_ *via* photoinduced hydrogen transfer and leaving CO adsorbed on the active site.

Taken together, these experimental results supported a mechanistic scenario in which, under basic conditions, ethanol underwent photoinduced dehydrogenation on dispersed oxidic iron sites to form acetaldehyde, followed by homolytic C─C bond cleavage to generate a methyl radical, which then recombined with surface‐bound hydrogen species to yield methane (Figure 5). Here, CO_2_ played a critical role by stabilizing iron redox cycling and suppressing unproductive hydrogen evolution, enabling sustained catalytic turnover. In contrast to conventional thermal decarbonylation processes that rely on low‐valent metal–carbonyl intermediates, the present system operated via photoinduced redox cycling at oxidic iron centers interfaced with a semiconductor support.

## 3 Conclusion

We have demonstrated a heterogeneous photocatalytic system that enables the dehydrogenative decarbonylation of primary alcohols under visible light using dispersed oxidic iron sites supported on aryl‐modified graphitic carbon nitride (Fe@f‐gC_3_N_4_). The catalyst couples alcohol oxidation and C─C bond cleavage within a single photoinduced redox cycle, producing one‐carbon‐shorter alkanes under mild conditions. Mechanistic studies established that methane formation originated exclusively from the alcohol substrate. At the same time, CO_2_ did not contribute carbon but instead stabilized the catalytic cycle by modulating the redox state of the iron centers and suppressing unproductive hydrogen evolution. Together, these results demonstrated that oxidic, earth‐abundant iron species can orchestrate complex multi‐bond transformations when embedded in an appropriately engineered photocatalytic framework, providing a general strategy for the light‐driven upgrading of renewable alcohols into hydrocarbons.

## Author Contributions

**Jun Hu**: investigation, writing – original draft, methodology, validation, data curation. **Wenhao Su**: investigation, methodology, validation. **Chaoqin Zeng**: investigation, methodology, validation, writing – original draft. **Bruno Vinicius Manzolli Rodrigues**: investigation, writing – original draft, methodology, validation. **Nils Rockstroh**: investigation, writing – original draft, methodology, validation. **Susanna Monti**: investigation, writing – original draft, methodology, validation. **Giovanni Barcaro**: investigation, writing – original draft, methodology, validation. **Piotr Kuśtrowski**: investigation, writing – original draft, methodology, validation. **Aleksander Jaworski**: investigation, writing – original draft, methodology, validation. **Jabor Rabeah**: investigation, writing – original draft, methodology, validation. **Adam Slabon**: investigation, writing – original draft, methodology, validation. **Shoubhik Das**: conceptualization, supervision, writing – review and editing.

## Conflicts of Interest

The authors declare no conflicts of interest.

## Supporting information




**Supporting File**: anie73782‐sup‐0001‐SuppMat.docx.

## Data Availability

The data that support the findings of this study are available from the corresponding author upon reasonable request.
