# Supplementary material for: CO2 as a Redox Shuttle Enables Photocatalytic Dehydrogenative Decarbonylation of Biomass‐Derived Alcohols to Light Alkanes
Source: Angew Chem Int Ed Engl. 2026 Jul 24;65(39):e9277722. doi: 10.1002/anie.9277722 (PMC13592308; doi:10.1002/anie.9277722)
Supplement: Supplementary file 1 [file ANIE-65-e9277722-s001.docx]

**Supporting information**

**CO_2_ as A Redox Shuttle Enables Photocatalytic Dehydrogenative Decarbonylation of Biomass derived Alcohols to Light Alkanes**

Jun Hu,^a^ Wenhao Su,^a^ Chaoqin Zeng,^b,c^ Bruno Vinicius Manzolli Rodrigues,^d^ Nils Rockstroh,^e^ Susanna Monti,^f^ Giovanni Barcaro,^g^ Piotr Kuśtrowski,^h^ Aleksander Jaworski,^i^ Jabor Rabeah,^b,e^ Adam Slabon,^d^ Shoubhik Das^a,*^

[a]	J. Hu, W. Su, and Prof. S. Das
Department of Chemistry
University of Bayreuth
Universitätstraße 30, 95447 Bayreuth, Germany
E-mail: Shoubhik.Das@uni-bayreuth.de

[b]	C. Zeng and Prof. J. Rabeah
State Key Laboratory of Low Carbon Catalysis and Carbon Dioxide Utilization, Lanzhou Institute of Chemical Physics Chinese Academy of Sciences
730000 Lanzhou, Gansu, China

[c]	C. Zeng
University of Chinese Academy of Science
Beijing 100049, China

[d]	Dr. B.V.M. Rodrigues, Prof. A. Slabon
Chair of Inorganic Chemistry
University of Wuppertal
Gaußstraße 20, 42119 Wuppertal, Germany

[e]	Prof. J. Rabeah and Dr. N. Rockstroh
Leibniz-Institut für Katalyse e.V. (LIKAT Rostock)
Albert-Einstein-Straße 29a, 18059 Rostock, Germany

[f]	Dr. S. Monti
CNR-ICCOM
Institute of Chemistry of Organometallic Compounds
Area della Ricerca, Pisa I-56124, Italy

[g]	Dr. G. Barcaro
CNR-IPCF
Institute for Chemical and Physical Processes
Area della Ricerca, Pisa I-56124, Italy

[h]	Prof. P. Kuśtrowski
Faculty of Chemistry
Jagiellonian University
Gronostajowa 2, 30-387 Kraków, Poland

[i]	Prof. A. Jaworski
Department of Chemistry
Stockholm University
Stockholm 10691, Sweden

Table of Contents

[S.1 General information	3](#_Toc232195680)

[S.2 Synthesis and characterization of the catalyst	3](#_Toc232195681)

[S.2.1 STEM and EDX	4](#_Toc232195682)

[S.2.2 X-ray photoelectron spectroscopy (XPS)	5](#_Toc232195683)

[S.2.3 Solid-state nuclear magnetic resonance (ssNMR)	5](#_Toc232195684)

[S.2.4 XANES and EXAFS	6](#_Toc232195685)

[S.2.5 Electron paramagnetic resonance	6](#_Toc232195686)

[S.2.6 Fourier-transform infrared (FTIR) experiments	6](#_Toc232195687)

[S.2.7 UV-vis and Mott-Schottky experiments	6](#_Toc232195688)

[S.3 Standard reaction procedure	8](#_Toc232195689)

[S.4 Apparent quantum efficiency (AQE), turnover frequency (TOF), or turnover number (TON)	8](#_Toc232195690)

[S.5 DFT calculations	9](#_Toc232195691)

[S.6 Supporting materials	11](#_Toc232195692)

[S.7 References	19](#_Toc232195693)

# S.1 General information

**Materials**: Chemicals and solvents were obtained from commercial suppliers (Sigma Aldrich, TCI, Thermo Fisher Scientific, J&K Scientific, Fluorochem and ABCR) and used without further purification. Non-commercial starting materials were prepared as described below according to literature procedures. All reactions were carried out under nitrogen atmospheres using Schlenck techniques. Thin Layer Chromatography (TLC) was performed using TLC plates from Merck (SiO_2_, Kieselgel 60 F254 neutral, on aluminum with fluorescence indicator) and compounds were visualized by UV (254 nm). Flash column chromatographic purification of products was accomplished using an automated chromatography system with on-line UV and ELSD detection using Grace® Silica flash Cartridges.

**NMR spectroscopy**: Nuclear Magnetic Resonance (NMR) spectra were recorded on a Bruker Avance III 400 (101 MHz for ^13^C) Fourier Transform NMR spectrometer at 300 K, using the non or partly deuterated solvent as internal standard (^1^H: δ = 7.26 ppm, ^13^C: δ = 77.16 ppm for CDCl_3_; ^1^H: δ = 2.50 ppm, ^13^C: δ = 39.52 ppm for DMSO-d_6_). Chemical shifts (δ) are given in ppm and coupling constants (*J*) are reported in Hertz (Hz). Multiplicities are described as s (singlet), d (doublet), t (triplet), q (quartet), p (pentet), br s (broad singlet) and m (multiplet) or combinations thereof. ^13^C NMR spectra were recorded with complete proton decoupling. ^1^H NMR yields were determined by the addition of a known amount of an internal standard and dissolving everything in a suitable deuterated solvent, followed by ^1^H NMR analysis.

**GC-MS**: Gas chromatography-mass spectrometry (GC-MS) samples were prepared by dissolving 0.1-5 mg of the compound in acetone (GC-MS quality) and further diluted to a concentration of 10^-5^ - 10^-6^ M. The samples were filtered using a CHROMAFIL® PET-20/25 syringe filter and 3 μL injected into the GC-MS (Agilent Technologies 7890 A GC System coupled to an Agilent Technologies 5975 C inert MSD with triple-axis detector). An Optima 725820.30 30 m × 250 μm × 0.25 μm column was used. The carrier gas was helium. Inlet temperature heater: 225 °C. Oven program: 70 °C for 3 min, then heating 5 K min^-1^ to 160 °C and heating at 160 °C for 2 min. A one-minute post-run at 280 °C ended the oven program.

**HRMS**: Samples for high-resolution mass spectrometry (HRMS) were prepared by diluting 0.1 mL of the reaction mixture to a final concentration of 10⁻⁵–10⁻⁶ M using ethyl acetate or acetonitrile. HRMS measurements were performed on a Q Exactive Orbitrap mass spectrometer (Thermo Fisher Scientific) equipped with an ESI source.

# S.2 Synthesis and characterization of the catalyst

**Figure S1**. The preparation procedure of Fe photocatalyst

107 mmol of Dicyandiamide (DCDA, 9 g), 0.08 mmol of Fe(NO_3_)_3_•9H_2_O (0.032 g), and 0.8 mmol of 2-amino-5-(trifluoromethyl)benzonitrile (0.150 g) were stirred together in 45 mL of H_2_O at 95 °C for 1 h in a closed system and then dried at 100 °C to remove water. Afterwards, solids were ground to fine powders and heated in a tubular furnace to 550 °C at a rate of 2.2 K /min and were kept at this temperature for 4 h under an aerobic atmosphere. After that, the oven was cooled down to room temperature, the resulting mixture was removed and ground in an agate mortar and used for the subsequent reactions. The as synthesized catalyst was named Fe@f-gC_3_N_4_.

## S.2.1 STEM and EDX

Scanning transmission electron microscopy (STEM) measurements were conducted at a probe aberration-corrected JEM-ARM200F (JEOL, Corrector: CEOS) operating at 200 kV. Energy-dispersive X-ray (EDX) spectroscopy was performed with a JED-2300 (JEOL) EDX spectrometer having a silicon drift detector (dry SD60GV). For STEM imaging, both High-Angle Annular Dark Field (HAADF) and Annular Bright Field (ABF) detectors were employed. The solid samples were dryly deposited onto a holey carbon-supported Cu grid (mesh 300) without any pre-treatment and subsequently transferred to the microscope for investigation.

**Figure S2**. STEM-HAADF image of Fe@f-gC_3_N_4_ (A), and selected EDX spectra of Fe@f-gC_3_N_4_ (B) of the regions highlighted by rectangles in (A).

**Figure S3**. STEM-HAADF image of Fe@f-gC_3_N_4_ after reaction (A), and selected EDX spectra of Fe@f-gC_3_N_4_ after one catalytic run (B) of the regions highlighted by rectangles in (A).

## S.2.2 X-ray photoelectron spectroscopy (XPS)

X-ray photoelectron spectroscopy analyses were carried out using a Prevac spectrometer equipped with a VG SCIENTA R3000 hemispherical analyzer. Spectra were collected with monochromatic Al Kα radiation (hν = 1486.6 eV) at a constant pass energy of 100 eV. Binding energy calibration was performed by setting the C 1s signal of adventitious carbon to 284.8 eV. Peak fitting was conducted in CasaXPS software after subtraction of a Shirley background, using mixed Gaussian-Lorentzian peak shapes with a GL(30) ratio, except for the component assigned to metallic Fe in the Fe 2p region, which was fitted using an asymmetric line shape.

## S.2.3 Solid-state nuclear magnetic resonance (ssNMR)

Magic-angle-spinning (MAS) NMR experiments were performed at a magnetic field of 14.1 T (Larmor frequencies of 600.12, 150.92, 60.83, and 564.69 MHz for ^1^H, ^13^C, ^15^N, and ^19^F respectively) on a Bruker Avance-III spectrometer. The ^1^H and ^19^F MAS NMR spectra were acquired using a 1.3 mm probehead and a 60 kHz MAS rate. This acquisition involved a use of a rotor-synchronized, double-adiabatic spin-echo sequence with a 90° excitation pulse of 1.25 µs (2.30 µs for ^19^F) followed by a pair of 50.0 µs tanh/tan short high-power adiabatic pulses (SHAPs) with 5 MHz frequency sweep. All pulses operated at the nutation frequency of 200 kHz (110 kHz for ^19^F). 128 signal transients (16384 for ^19^F) were acquired. Cross-polarization (CP) ^1^H-^13^C and ^1^H-^15^N CPMAS NMR spectra were recorded using a 7 mm probehead with a 7 kHz MAS rate and 65 kHz spinal64 proton decoupling. For ^1^H-^13^C CPMAS acquisition Hartmann-Hahn matched radiofrequency fields were applied for a contact interval of 1.5 ms and 1024 signal transients were collected using a relaxation delay of 5 s. The ^1^H-^15^N CPMAS acquisitions involved contact interval of 5ms, and 16384 scans collected with relaxation time of 5 s. Chemical shifts are reported with respect to tetramethylsilane (^1^H, ^13^C), nitromethane (^15^N), and trichlorofluoromethane (^19^F).

## S.2.4 XANES and EXAFS

Extended X-ray absorption fine structure (EXAFS) experiments were carried out at the beamline 10 of the DELTA storage ring (TU Dortmund, Germany), operating with 1.5 GeV electrons and a stored current of 100–130 mA (energy range: 4–14 keV). The incident beam was monochromatized using a Si(311) double crystal monochromator, detuned by 30 % to suppress higher harmonics and ensure beam stability. The beam size at the sample position was defined by slits to 3 mm (horizontal) x 0.4 mm (vertical). Measurements were conducted in transmission mode using argon-filled ionization chambers, adjusted to achieve an absorption of approximately 25-45% in the first chamber and 50-75% in the chamber behind the sample. Energy calibration was performed using an Fe foil reference. Data processing and analysis were performed using the *Demeter^[1]^* software package.

## S.2.5 Electron paramagnetic resonance

Fe@f-gC₃N_4_ (5 mg) and KOH (20 mg) were placed in a flat quartz EPR cell, which was thoroughly purged with argon to remove residual oxygen. Separately, a solvent mixture was prepared by first treating a small amount of KOH with anhydrous ethanol to minimize residual water, followed by addition of acetonitrile. This solution was also purged with argon to ensure oxygen-free conditions. While maintaining a continuous argon flow over the flat cell, a small volume of the prepared solvent was introduced, followed by 10 μL of DMPO as the spin-trapping agent. All manipulations were conducted under an inert argon atmosphere to prevent oxygen contamination. After recording the initial EPR spectrum, CO₂ was introduced into both the flat cell and the solvent under strictly oxygen-free conditions, and the EPR measurement was repeated.

## S.2.6 Fourier-transform infrared (FTIR) experiments

**First reaction:** 4 mg of Fe@f-gC₃N₄ catalyst was added to 4 mL of CH₃CN containing 0.1 mL of CH₃CH₂OH, and the mixture was irradiated with a 390–400 nm LED light source for 11 hours under a CO₂ atmosphere.

**Second reaction:** 4 mg of Fe@f-gC₃N₄ catalyst and 26.9 mg of KOH were mixed in 4 mL of CH₃CN containing 0.1 mL of CH₃CH₂OH, and the mixture was irradiated with a 390–400 nm LED light source for 11 hours under a CO₂ atmosphere.

After each reaction, approximately 1 mg of the solid was thoroughly mixed with about 100 mg of dried spectroscopic-grade potassium bromide (KBr) and finely ground in an agate mortar. Each mixture was then pressed into a transparent pellet under a certain pressure. The pellet was subsequently measured using a Bruker Invenio FTIR instrument.

## S.2.7 UV-vis and Mott-Schottky experiments

The solid UV–Vis absorption curve (**Figure** **S4**) was obtained from diffuse reflectance spectra (DRS) recorded on a Shimadzu UV-3101PC spectrophotometer. BaSO_4_ was used as the reflectance standard. Ultraviolet-visible diffuse reflectance spectroscopy (UV-vis DRS) was employed to investigate the electronic band structure. The optical band gap, estimated from the Tauc plot, was found to be approximately 2.50 eV. Band gap of reference materials are also shown in **Figure S4**.

**Figure S4**. Band gap and redox potentials of g-C_3_N_4_, Fe@g-C_3_N_4_, f-gC_3_N_4_, and Fe@f-gC_3_N_4_.

The Mott-Schottky experiments (**Figure** **S5**) were conducted using a three-electrode configuration cell. The working electrode consisted of a thin film obtained through the drop-coating method, while the counter electrode comprised a Pt-foil electrode, and the reference electrode comprised Ag/AgCl/3M KCl. The thin film electrodes were prepared by combining 5 mg of the sample with a solution composed of 50 μL ethanol and 10 μL of 5 wt% Nafion solution, followed by sonication for 1 hour. Subsequently, 15 μL of the resulting mixture was pipetted onto a FTO glass surface to create 1 × 1 cm² coating films, which were then dried at room temperature and further subjected to overnight drying at 40 °C in a vacuum oven. The electrolyte solution employed was 0.5 M Na_2_SO_4_ (with a measured pH of 7.47), degassed with N_2_. Measurements were carried out using a Metrohm Autolab potentiostat-galvanostat PGSTAT204 workstation in the absence of light, with an AC amplitude of 5 mV and frequency of 1000 Hz.

To further clarify the electronic structure, electrochemical flat-band potential measurements were conducted. Typically, flat-band potential values were derived from the Mott-Schottky equation.

$$\begin{aligned} \frac{1}{C^{2}}=\frac{2}{\varepsilon\varepsilon_{0}N_{D}}(E-E_{fb}-\frac{K_{B}T}{q})\#(1) \#\# \end{aligned}$$

Here, C represents the space charge capacitance, N_D_ denotes the donor density, and ε, ε_0_ are the dielectric constants of the film electrode and free space, respectively. E refers to the applied potential, E_fb_ is the flat-band potential, K_B_ is Boltzmann’s constant, T is the temperature, and q is the electronic charge. The flat-band potential E_fb_ can be obtained by extrapolating the plot to where 1/C^2^ = 0 (**Figure S5**).

The positive slope of the Mott-Schottky curve suggests that the photocatalyst is n-type. Consequently, the flat band potential (E_fb_) of Fe@f-gC_3_N_4_ is approximately -1.08 V (vs Ag/AgCl (3M KCl), as determined from the intersection between the Mott-Schottky plot and the baseline. In *n*-type semiconductor photocatalyst, the conduction band generally lies closely to the flat-band potential, with this offset influenced by factors such as electron effective mass and carrier concentration. So, the conduction band of Fe@f-gC_3_N_4_ can be calculated as -1.08 V (vs. Ag/AgCl (3M KCl)). Additionally, using the band gap energy E_g_, the valence band (VB) can be determined by E_VB_=E_CB_+E_g_. Therefore, the valence band (VB) of Fe@f-gC_3_N_4_ was at ca. +1.42 V (vs. Ag/AgCl (3M KCl)). Based on: E_RHE_=E_Ag/AgCl (3M KCl)_ + 0.21 + (0.059 x 7.47) V, so E_CB_ = -0.43 V (vs RHE), E_VB_= +2.07 V (vs SCE).

**Figure S5**. Mott-Schottky plots of g-C_3_N_4_, Fe@g-C_3_N_4_, f-gC_3_N_4_, and Fe@f-gC_3_N_4_.

# S.3 Standard reaction procedure

All photocatalytic experiments were conducted in 30 mL Schlenk tubes under ambient temperature. In a typical reaction, the reactor was charged with the Fe@f-gC_3_N_4_ photocatalyst (4 mg), KOH (0.12 M final concentration), and a magnetic stirring bar. The tube was sealed with a rubber septum and evacuated for 30 minutes to remove residual air, followed by backfilling with CO_2_ gas. Subsequently, 4 mL of anhydrous acetonitrile and 0.1 mL of alcohol were introduced into the reactor via syringe. To maintain a constant pressure of the reactant gas, a balloon filled with CO_2_ was attached to the vessel using a needle through the septum. The reaction mixture was stirred and subjected to visible-light irradiation using a 390 nm LED lamp for a duration of 18 hours. Upon completion of the reaction, the gaseous products in the headspace were collected and quantified via gas chromatography-mass spectrometry (GC-MS) using a headspace sampling technique.

# S.4 Apparent quantum efficiency (AQE), turnover frequency (TOF), or turnover number (TON)

The apparent quantum yield of methane in 0.12M KOH acetonitrile solution with 0.1 mL ethanol under CO_2_ in 18h was calculated as following.

The amount of formed product (mol): M = 2.41 * 10^-5^ mol. The light intensity of Kessil LED lamp (λ = 390 nm, 40W) was 0.12 W/cm^2^.

$$\eta=\frac{N_{e}}{N_{p}}\times100\%=\frac{2\times M\times N_{A}}{\frac{S\times P\times t}{h\times\frac{c}{\lambda}}}\times100\%=\frac{2\times M\times N_{A}\times h\times c}{S\times P\times t\times\lambda}\times100\%=\frac{2*2.41\times{10}^{-5}*6.022\times{10}^{23}\times6.63\times{10}^{-34}\times3\times{10}^{8}}{6.0\times\pi\times0.12\times18\times3600\times390\times{10}^{-9}}\times100\%=0.01\%$$

Where, M represents the amount of formed product (mol), N_A_ is the Avogadro constant (6.022x10^23^ mol^-1^ ), h is the Planck constant (6.63x10^-34^ J·s), c is the light speed (3x10^8^ m·s^-1^), S is the irradiation area (6.0π cm^2^), P is the light intensity (0.12 W·cm^-2^), t is irradiation time (18 x 3600 s), and λ is the wavelength of light (m).

$$TON=\frac{Moles of {CH}_{4} produced}{Total moles of Fe in the catalyst}=\frac{2.41\times{10}^{-5} mol}{\frac{0.145 wt\%\times4.0\times{10}^{-3} g}{55.845 g/mol}}=232.045$$

$$TOF=\frac{TON}{Reaction time}=\frac{232045}{18 h}=12.891 h^{-1}$$

# S.5 DFT calculations

The Gaussian 16 program^[2]^ was used for optimizing the molecular adducts in acetonitrile solution (in agreement with the experiments) at the density functional theory (DFT) level with the B3LYP hybrid functional, the Grimme DFT-D3(BJ) empirical dispersion^[3]^, the 6-31G(d,p) basis set for the C, N, O, F, and H atoms, and the def2-TZVPD basis set for the metal center (Fe). Acetonitrile (ε = 35.69) was described as a continuum using the SMD approach.

Possible reaction pathways and activation energy barriers (saddle points) were identified using the nudged elastic band (NEB) method in the Quantum Espresso package^[4]^. Projector Augmented-Wave (PAW) pseudopotentials and the generalized gradient approximation (GGA) with the Perdew–Burke–Ernzerhof (PBE) functional were used. Dispersion corrections were added according to the Grimme-D3 parametrization implemented in the QE package. Wave-function and charge-density cutoffs of 50 and 500 Ry were employed for the plane-wave basis sets. Single-particle wave functions were calculated spin-unrestricted by applying a smearing of the one-particle levels of 0.002 Ry. The NEB calculations were performed using the optimized geometries obtained with Gaussian16 as reactants and products. The identified saddle points were optimized at the transition-state level using Gaussian software, as for the minimum-energy configurations.

**Figure S6. Top:** Complex system initially simulated to produce possible starting configurations of the experimental setup. **Bottom left**: zoomed portion of the catalyst where an ethanol molecule (ball and stick representation) is adsorbed on Fe and surrounded by solvent (acetonitrile) and CO_2_ molecules. **Bottom right**: reduced model chosen for the quantum-chemistry optimizations (optimized configuration of the reactants). Color codes: O red, N blue, C, grey, F green, H white, Fe orange.

# S.6 Supporting materials

**Table S1**. Control experiments of dehydrogenative decarbonylation of ethanol.

| Entry | Note | CH_4_ (μmol g^-1^ h^-1^) | | C_2_H_4_ (μmol g^-1^ h^-1^) | CO (μmol g^-1^ h^-1^) |
| --- | --- | --- | --- | --- | --- |
| 1 | No change | 334.7 | 1.4 | | 61.3 |
| 2 | No EtOH | 0 | 0 | | 0 |
| 3 | No MeCN | 115.0 | 3.2 | | 6.8 |
| 4 | No light (40 °C) | 0 | 0 | | 0 |
| 5 | No catalyst | 0 | 0 | | 0 |
| 6 | Acetaldehyde | 696.2 | 0 | | 56 |
| 7 | Acetic acid | 702.4 | 0 | | 26 |
| 8 | Acetaldehyde, no catalyst | 86.1 | 0 | | 8 |
| 9 | Acetic acid, no catalyst | 1.5 | 0 | | 0 |

To further evaluate the proposed reaction pathway, acetaldehyde and acetic acid were independently examined as substrates under the standard photocatalytic conditions. Acetaldehyde afforded methane at a rate of 696 μmol g^-1^ h^-1^ together with minor amounts of CO and ethene, confirming that it is readily converted under the reaction conditions. Considering that acetaldehyde was directly detected during ethanol conversion, these results strongly support its role as a key intermediate in the dehydrogenative decarbonylation pathway. Acetic acid also underwent conversion to methane, CO, and ethene, demonstrating that the catalyst is capable of promoting transformations of carboxylic acids. However, no acetic acid was detected during ethanol conversion by either GC-FID or NMR analysis. Therefore, although acetic acid is a competent substrate, the available evidence does not support its participation as a major intermediate in the ethanol-to-methane pathway. These observations are consistent with a mechanism involving initial dehydrogenation of ethanol to acetaldehyde, followed by C–C bond cleavage and subsequent reduction steps leading to methane formation.

**Table S2**. Production rate of dehydrogenative decarbonylation reaction using mixed alcohols.

| Alcohols | Production rate (μmol g^-1^ h^-1^) | | | | |
| --- | --- | --- | --- | --- | --- |
|  | Methane | Ethane | Propane | Butane | Others |
| Ethanol + Propanol | 111.7 | 33.8 | 0 | 0 |  |
| Butanol + Pentanol | 17.3 | 5.7 | 36.4 | 13.2 |  |
| Ethylene glycol | 3.0 | 1.3 | 0 | 0 | 0.7 (Methanol) |
| Glycerol | 2.1 | 0.5 | 0 | 0 | 0.5 (Ethylene glycol) |

**Figure S7**. GC-FID spectra of reactions mixture. Reference spectrum (up), reaction under CO_2_ atmosphere (bottom left), reaction under O_2_/air atmosphere (bottom right).

**Figure S8**. NMR spectra of reactions mixture. ^1^H spectrum (left), and ^13^C spectrum (right).

**Figure S9**. GC-MS spectra of reaction with TEMPO.

**Figure S10**. GC-MS spectra of reaction with diphenylethylene.

**Figure S11**. Head-Space GC-MS spectrum of reaction (methane peak is presented).

**Figure S12**. Head-Space GC-MS spectrum of reaction (carbon monoxide peak is presented).

**Figure S13**. Time resolved EPR spectra of Fe@f-gC_3_N_4_ catalyst.

**Figure S14**. EPR/simulated EPR spectra of carbon centered radical before and after introducing CO_2_.

**Figure S15:** FTIR spectra of the catalsyst systems without/with KOH.

**Figure S16:** XPS spectra of fresh Fe@f-gC₃N₄ and KOH-treated Fe@f-gC₃N₄ recorded in the Fe 2p, O 1s, and C 1s regions. The KOH-treated sample was prepared by exposing the catalyst to a KOH-containing acetonitrile/ethanol solution under inert atmosphere, followed by washing and vacuum drying. The spectra show the presence of residual KOH-derived surface species after treatment, while no significant changes are observed in the Fe 2p region.

**Figure S17:** Normalized Fe K-edge XANES spectra and first-derivative XANES spectra of fresh Fe@f-gC₃N₄ and KOH-treated Fe@f-gC₃N₄. The KOH-treated sample was prepared by exposing the catalyst to a KOH-containing acetonitrile/ethanol solution under inert atmosphere, followed by washing and vacuum drying. The spectra show no significant changes in the edge position.

# S.7 References

[1]	B. Ravel, M. Newville, *J. Synchrotron Rad.* **2005**, *12*, 537-541.

[2]	M. J. Frisch, G. W. Trucks, H. B. Schlegel, G. E. Scuseria, M. A. Robb, J. R. Cheeseman, G. Scalmani, V. Barone, G. A. Petersson, H. Nakatsuji, X. Li, M. Caricato, A. V. Marenich, J. Bloino, B. G. Janesko, R. Gomperts, B. Mennucci, H. P. Hratchian, J. V. Ortiz, A. F. Izmaylov, J. L. Sonnenberg, Williams, F. Ding, F. Lipparini, F. Egidi, J. Goings, B. Peng, A. Petrone, T. Henderson, D. Ranasinghe, V. G. Zakrzewski, J. Gao, N. Rega, G. Zheng, W. Liang, M. Hada, M. Ehara, K. Toyota, R. Fukuda, J. Hasegawa, M. Ishida, T. Nakajima, Y. Honda, O. Kitao, H. Nakai, T. Vreven, K. Throssell, J. A. Montgomery Jr., J. E. Peralta, F. Ogliaro, M. J. Bearpark, J. J. Heyd, E. N. Brothers, K. N. Kudin, V. N. Staroverov, T. A. Keith, R. Kobayashi, J. Normand, K. Raghavachari, A. P. Rendell, J. C. Burant, S. S. Iyengar, J. Tomasi, M. Cossi, J. M. Millam, M. Klene, C. Adamo, R. Cammi, J. W. Ochterski, R. L. Martin, K. Morokuma, O. Farkas, J. B. Foresman, D. J. Fox, Wallingford, CT, **2016**.

[3]	S. Grimme, S. Ehrlich, L. Goerigk, *J. Comput. Chem.* **2011**, *32*, 1456-1465.

[4]	P. Giannozzi, O. Andreussi, T. Brumme, O. Bunau, M. Buongiorno Nardelli, M. Calandra, R. Car, C. Cavazzoni, D. Ceresoli, M. J. J. o. p. C. m. Cococcioni, *J. Phys.: Condens. Matter* **2017**, *29*, 465901.
